# Mechanistic insights into the detection of free fatty and bile acids by ileal glucagon-like peptide-1 secreting cells

**DOI:** 10.1016/j.molmet.2017.11.005

**Published:** 2017-11-11

**Authors:** Deborah A. Goldspink, Van B. Lu, Lawrence J. Billing, Pierre Larraufie, Gwen Tolhurst, Fiona M. Gribble, Frank Reimann

**Affiliations:** Metabolic Research Laboratories and MRC Metabolic Diseases Unit, Institute of Metabolic Science, Addenbrooke's Hospital, Hills Road, Cambridge CB2 0QQ, UK

**Keywords:** GLP-1, FFA1, GPBAR1, Organoid, Diabetes, Obesity

## Abstract

**Objectives:**

The aim of this study was to investigate the electrical properties of ileal Glucagon-like peptide 1 (GLP-1) secreting L-cells using murine organoid cultures and the electrophysiological and intracellular signaling pathways recruited following activation of the G_αq_-coupled free fatty acid receptors FFA1 and G_αs_-coupled bile acid receptors GPBAR1.

**Methods:**

Experiments were performed using ileal organoids generated from mice transgenically expressing fluorescent reporters (Epac2-camps and GCaMP3) under control of the proglucagon promoter. Electrophysiology and single cell imaging were performed on identified L-cells in organoids, and GLP-1 secretion from cultured organoids was measured by immunoassay.

**Results:**

The FFA1 ligand TAK-875 triggered L-cell electrical activity, increased intracellular calcium, and activated a depolarizing current that was blocked by the TRPC3 inhibitor Pyr3. TAK-875 triggered GLP-1 secretion was Pyr3 sensitive, suggesting that the TRPC3 channel links FFA1 activation to calcium elevation and GLP-1 release in L-cells. GPBAR1 agonist triggered PKA-dependent L-type Ca^2+^ current activation and action potential firing in L-cells. The combination of TAK-875 and a GPBAR1 agonist triggered synergistic calcium elevation and GLP-1 secretory responses.

**Conclusions:**

FFA1 and GPBAR1 activation individually increased electrical activity in L-cells by recruiting pathways that include activation of TRPC3 and L-type voltage-gated Ca^2+^ channels. Synergy between the pathways activated downstream of these receptors was observed both at the level of Ca^2+^ elevation and GLP-1 secretion.

## Highlights

• Organoid-derived ileal L-cells recapitulate the properties of primary ileal L-cells.
• GPBAR1 agonism stimulates L-type Ca^2+^ current-dependent action potentials in L-cells.
• FFA1 agonists depolarize L-cells via TRPC3 activation.
• Synergy between GPBAR1/FFA1 occurs at the levels of Ca^2+^ responses and secretion.

## Graphical abstract

**Figure:**
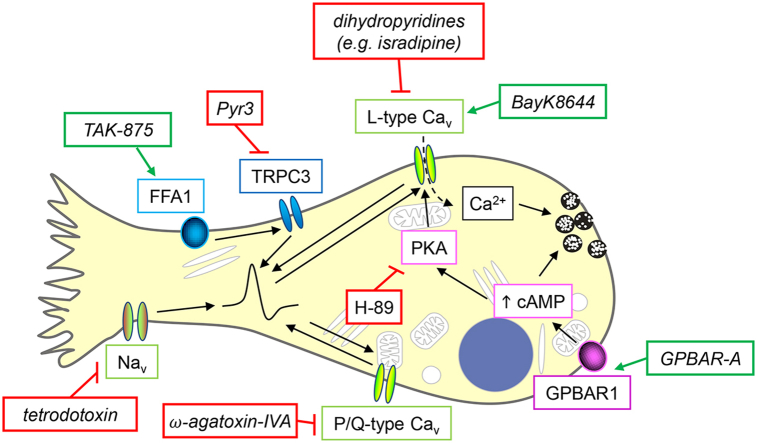

## 1 Introduction

Enteroendocrine cells (EECs) are found scattered along the gastrointestinal tract and produce hormones that dynamically link metabolism and appetite to rates of nutrient absorption. Enteroendocrine L-cells, for example, produce several hormones with demonstrated or potential translational impact, including Glucagon-like peptide-1 (GLP-1), which enhances insulin secretion and satiety [1], [2], GLP-2, which promotes intestinal growth, and Peptide YY (PYY), which reduces appetite. There is considerable interest in developing pharmacological agents that target L-cell secretion, with the prediction that they will mimic some of the beneficial effects of gastric bypass surgery on type 2 diabetes and obesity, believed to result, at least in part, from elevated GLP-1 and PYY levels [3]. A better understanding of the molecular mechanisms underlying stimulus detection and integration in L-cells would critically benefit this therapeutic approach.

Physiological release of GLP-1 is stimulated by the local absorption of nutrients following their digestion in the gut lumen. L-cell glucose detection is mediated by the coupled uptake of Na^+^ ions with substrate by brush border sodium coupled glucose transporters (SGLT1) [4]. Luminal contents are also detected by G-protein coupled receptors (GPCRs) linked either to G_αs_ (e.g. bile acid detection by the G-protein coupled bile acid receptor GPBAR-1, also known as TGR5) or G_αq_ (e.g. long chain fatty acid detection by the free fatty acid receptor FFA1, also known as GPR40). Studies on the G_αs_-coupled receptor GPR119 concluded that its pharmacological activation was insufficient to exert a metabolic benefit in humans [5], but more recent studies have reported that concomitant activation of G_αs_ and G_αq_ pathways in L-cells is a much more effective stimulus of GLP-1 secretion than either pathway individually [6].

Studying signaling pathways in single L-cells is now possible using transgenic mouse models exhibiting cell-specific expression of fluorescent reporters and sensors of intracellular Ca^2+^ and cAMP [4], [7], [8], [9]. Transcriptomic analyses and primary intestinal cultures from these models have been used to identify and characterize a variety of detection pathways for nutrients, hormones, and local signaling molecules [10]. More recently, intestinal organoid cultures have permitted the growth and regeneration of intestinal epithelium in a 3-dimensional model system [11], and have been validated for monitoring enteroendocrine hormone secretion [12], [13], [14].

The objectives of this study were to identify the electrophysiological and second messenger responses to FFA1 and GPBAR1 activation in single L-cells. Intestinal organoids from transgenic mouse models expressing cell specific fluorescent sensors of cAMP and Ca^2+^ provided a consistent source of L-cells that were readily studied by electrophysiological methods.

## 2 Materials and methods

### 2.1 Glucagon reporter organoid lines and primary ileal cultures

This research has been regulated under the Animals (Scientific Procedures) Act 1986 Amendment Regulations 2012 following ethical review by the University of Cambridge Animal Welfare and Ethical Review Body (AWERB). Ileal intestinal organoid lines were established from mice expressing the GCaMP3 reporter in Cre expressing cells, with *Cre* expressed under the control of the proglucagon promoter [4], [8]. Ileal intestinal organoid lines were also established from mice expressing the FRET-based cAMP sensor *Epac2camps* under the control of the proglucagon promoter [9]. Organoid protocol was modified from Sato et al., 2009. Distal (last 10 cm) mouse small intestinal tissue (ileal) was opened and washed in ice cold PBS; the tissue was chopped into 3–5 mm pieces and then further washed in ice cold PBS. Tissue pieces were placed in ice cold 30 mM EDTA in PBS for 5 min, transferred to cold PBS and shaken vigorously for 20 s (fraction 1). EDTA treatment and subsequent PBS shaking was repeated 2 more times (fraction 2–3) followed by a further 2 times shaking in PBS alone (fractions 4–5). The fraction with the most crypts was selected after examination under a microscope, villi structures were removed by filtering through a 70 μm cell strainer (Thermo Fisher Scientific), and the remaining crypts were centrifuged at 200G for 5 min. The crypt pellet was resuspended in Matrigel (200 μl, Corning), and aliquots were polymerized at 37 °C for 30 min in 48-well plates (Nunc; 15 μl/well). Organoid medium [11] supplemented with 10 μM ROCK inhibitor y27632 (Tocris) was added to each well. Medium was changed every 2–3 days, with organoids passaged every 7 days as previously described [11]. Mixed primary ileal intestinal cultures were prepared as previously described [15].

### 2.2 2D organoid culture

For 2D culture, organoids were collected in ice cold Advanced DMEM:F12 (ADF) medium (Life Technologies) and centrifuged at 200G for 5 min. The organoid pellet was broken-up enzymatically with trypLE (Gibco) for 2 min at 37 °C, before being resuspended in ADF containing 10% FBS (Gibco) and 10 μM y27632. If necessary, organoids were further broken-up by trituration. Resulting single-cells and clusters were pelleted at 300G for 5 min, re-suspended in organoid medium (+10 μM y27632) and seeded onto 2% Matrigel coated glass bottom dishes (Matek) for imaging experiments, 48-well plates for GLP-1 secretion measurement or plastic dishes for electrophysiology experiments.

### 2.3 Expression analysis of L-cell population

RNA sequencing (n = 3 mice) of FACS-purified L-cells from the ileum and colon of Glu-Venus mice was performed as described previously [16]. All sequencing was performed at the Transcriptomics and Genomics Core Facility (Cancer Research UK Cambridge Institute) using an Ilumina HiSeq 2500 system.

### 2.4 GLP-1 secretion

For GLP-1 secretion experiments ileal-derived organoids were seeded into 48-well plates as described above. 1–2 days following seeding, 2D cultures were washed 3 times in warm 138 buffer containing 1 mM glucose and 0.1% fatty acid–free BSA. Cells were incubated for 20 min in 1 mM glucose in 138-buffer at 37 °C, which was completely removed before test agents dissolved in 150 μl of the same buffer were added and incubated at 37 °C for 2 h. Supernatants were removed from the organoids and spun at 350G for 5 min at 4 °C, transferred to a fresh tube and snap frozen on dry ice. Meanwhile, the cells were lysed in 150 μl of lysis buffer on ice for 30 min. Lysates were scraped and collected, followed by centrifugation at 8000G for 10 min at 4 °C, and resulting supernatants snap frozen until measurement. GLP-1 levels were measured using the total GLP-1 ELISA kit (MesoScale) as per manufacturer instruction. GLP-1 secretion was calculated first as a percentage of individual well content and second as fold change in comparison to wells treated with 138 buffer without additions in parallel on each plate (basal, containing 1 mM glucose and 0.1% BSA). To examine potential synergy between FFA1 and GPBAR1 pathways, we added the % of GLP-1 released of single drug treatments (above basal conditions) to give a predicted % of GLP-1 secretion and subtracted this from the observed % of GLP-1 secreted by the simultaneous application of both drugs.

### 2.5 Immunohistochemistry

Organoids were retrieved from Matrigel using Cell Recovery Solution (Corning) and fixed in 4% paraformaldehyde in PBS (Alfa Aesar) for 30 min at room temperature. 2D cultures on glass coverslips were fixed in 4% paraformaldehyde in PBS for 20 min. Immunostaining was performed as previously described [17]. Rabbit polyclonal antibodies against proglucagon (Santa Cruz, sc-13091) were used at 1:200 and goat polyclonal antibodies against GFP (Abcam, ab5450) at 1:1000 to detect YFP and GCaMP3. Secondary antibodies conjugated to Alexa-Fluor 488 and 555 (Invitrogen) were used at 1:1000 and Hoescht (Sigma) nuclear stain at 1:3000.

### 2.6 Electrophysiology

Ileal organoids or primary ileal cultures were plated as a 2D monolayer 1–3 days prior to recording on 35 mm dishes. Experiments were performed on single cells or well-defined cells in small clusters. Membrane potential and currents were recorded in the perforated-patch configuration using an Axopatch 200B connected through a Digidata 1440A A/D converter and pCLAMP software (Axon Instruments). Microelectrodes were pulled from borosilicate glass (1.5 mm OD, 1.16 mm ID; Harvard Apparatus) and the tips coated with refined yellow beeswax. Electrodes were fire-polished using a microforge (Narishige) and had resistances of 2–3 MΩ when filled with pipette solution. A silver/AgCl ground wire connected to the bath solution via a 0.15 M NaCl agar bridge was used as a ground.

To trigger action potential firing, current was injected to maintain the cell at −70 mV and 10 ms current pulses, increasing in magnitude by 2 pA, was applied at 0.2 Hz. A peak threshold of −10 mV was used to positively identify an action potential for further analysis. The action potential peak was taken as the maximum voltage reached during the protocol described above. The threshold of action potential firing was determined as the voltage at which an action potential began its rapid upstroke. The width of the action potential waveform was measured at 50% of the action potential peak, or the action potential half-width. For pharmacological assessment of the action potential waveform, the action potential peak was measured after application of channel blocker and expressed as a % of total block by application of TTX (0.3 μM) + Cd^2+^ (100 μM).

To examine the pattern of action potential firing, current was injected to maintain the cell at −70 mV and 500 ms current steps, sequentially increasing in amplitude by 2 pA, were injected. The total number of action potentials that crossed a threshold of −10 mV elicited during the current injection was plotted against the magnitude of the current injected.

Inward currents and isolated Ca^2+^ currents were recorded by applying a series of 70 ms voltage steps from −110 mV to +60 mV, from a holding potential of −80 mV. Peak current from the fast or Na^+^ current component was determined as the minimum peak current occurring within 1 ms of the capacitative transient current. The peak current from the slow or Ca^2+^ current component was measured from the current 10 ms following the application of a voltage step. *I*_*Ca*_-voltage relationship was assessed with 160 ms voltage ramps, from a holding potential of −80 mV to +80 mV. Ten ramps were averaged per treatment for each cell and normalized to the baseline peak *I*_Ca_ amplitude.

To investigate hyperpolarization-activated currents (HCN), a series of 2 s voltage steps from −50 to −140 mV was applied from a holding potential of −50 mV. Current–voltage relationships were studied by repeatedly ramping the voltage over 500 ms from −100 to −50 mV, from a holding potential of −80 mV. Twenty ramps were averaged before and during application of GPBAR-A.

To measure currents elicited following application of the FFA1 agonist TAK-875, 120 ms voltage ramps from −120 to 0 mV, from a holding potential of −70 mV, were applied and at least ten ramps were averaged for each treatment per cell. Current amplitudes were measured at −90 mV, subtracted from baseline current measured before treatment (Δ current) and compared between treatments.

The inter-spike membrane potential (ISMP) was assessed by fitting a Gaussian curve to an “all-points” histogram of a 30 s recording of baseline or treatment. The mean or peak of the fitted curve was taken as ISMP.

### 2.7 Microscopy

Immunostained cells were imaged on an SP8 confocal microscope (Leica Microsystems). *In situ* organoids were imaged on an EVOS microscope (Thermo Fisher Scientific). Time-lapse microscopy of organoids was imaged using Incucyte Zoom imaging system (Essen BioScience). Images were processed using LAS-X (Leica), Photoshop (Adobe), and ImageJ (NIH) software. GCaMP3 and Epac2camps imaging experiments were performed as previously described using an inverted microscope and Metafluor software (Molecular Devices) [9], [15]. For GCaMP3 time-lapse microscopy, images were taken every 2 s. For fluorescence intensity analysis exclusive thresholding and background subtraction was first applied, mean whole cell fluorescence intensity was then calculated and a 30 s rolling average was generated for each trace. Changes in fluorescence intensity were measured as the difference in max intensity before and during treatment (ΔFI). For Epac2camps FRET time-lapse microscopy images were taken every 5 s, whole cell fluorescence intensity of both donor (CFP) and acceptor (YFP) was measured after exclusive thresholding and background subtraction was applied. A 30 s rolling average was generated for the ratio of CFP/YFP and changes in this ratio was measured as the difference in max ratio before and during treatment (ΔCFP/YFP). To determine whether the GCaMP3 response to co-application of FFA1 and GPBAR1 agonists was synergistic, we calculated for every cell what the additive increase in GCaMP3 fluorescence intensity should be (based on single treatment of each drug on that cell) and compared this to the actual GCaMP3 fluorescence intensity produced by co-application of both drugs.

### 2.8 Solutions, drugs and chemicals

Standard saline solution (138 buffer) used for secretion, imaging, and electrophysiological experiments consisted of (in mM) NaCl (138), KCl (4.5), HEPES (10), NaHCO_3_ (4.2), NaH_2_PO_4_ (1.2), d-glucose (1), CaCl_2_ (2.6), MgCl_2_ (1.2); pH 7.4 with NaOH. For experiments where a higher KCl concentration was used (30 mM), the standard saline solution was adjusted to replace Na^+^ with K^+^.

For perforated-patch recordings, the internal pipette solution contained (in mM) K_2_SO_4_ (76), NaCl (10), KCl (10), HEPES (10), sucrose (55), MgCl_2_ (1); pH 7.2 with KOH. Amphotericin B (2–2.5 μg/mL) dissolved in DMSO was added to the pipette filling solution on the day of recording.

For isolating inward currents, an external recording solution containing (in mM) NaCl (118), CsCl (5.6), HEPES (10), TEA-Cl (20), d-glucose (1), CaCl_2_ (5), pH 7.4 with NaOH, was used. For recording Ca^2+^ currents, the same external solution described above was applied with the addition of tetrodotoxin or TTX (0.3 μM). The same internal pipette solution for perforated-patch recordings was used to record inward currents and isolated Ca^2+^ currents with the exception of K_2_SO_4_, which was replaced with an equal concentration of Cs_2_SO_4_.

Lysis buffer used in secretion assays consisted of (in mM) Tris–HCl (50), NaCl (150), 1% IGEPAL-CA 630, 0.5% deoxycholic acid and supplemented with complete EDTA-free protease inhibitor cocktail (Roche).

Unless otherwise stated, all chemicals were purchased from Sigma–Aldrich. TTX, ω-agatoxin-IVA and nifedipine were purchased from Alomone Laboratories; TAK-875 and isradipine were purchased from Adooq Bioscience. Drug stocks were made as a 1000× concentrated stock and diluted to a final working concentration on the day of experiment. Drugs for imaging and electrophysiology experiments were applied directly onto cells using a custom-made gravity-fed perfusion system. A constant flow of external solution was applied onto cells during baseline recordings and switched to a drug solution during drug applications to avoid flow-induced artifacts. All recordings were performed at room temperature (20–24 °C).

### 2.9 Statistical analyses

Statistical tests were performed with GraphPad Prism 7 (GraphPad Software). Individual data points were represented on graphs with the mean ± S.E.M. or median ± interquartile range. Statistical significance between two groups was determined using an unpaired *t*-test, paired t-test or Wilcoxon matched-pairs signed rank test, as indicated. To compare three or more groups, a one-way analysis of variance (ANOVA) test followed by the indicated multi-comparison test was performed. *P* < 0.05 was considered to represent statistical significance. GLP-1 secretion analysis was performed on log transformed data. For synergy and fluorescence intensity/ratio change analysis, a one-sample t-test or a Wilcoxon signed-rank test was performed.

## 3 Results

### 3.1 Generation of organoids from transgenic mice expressing fluorescent labels in L-cells

**Figure 1:**
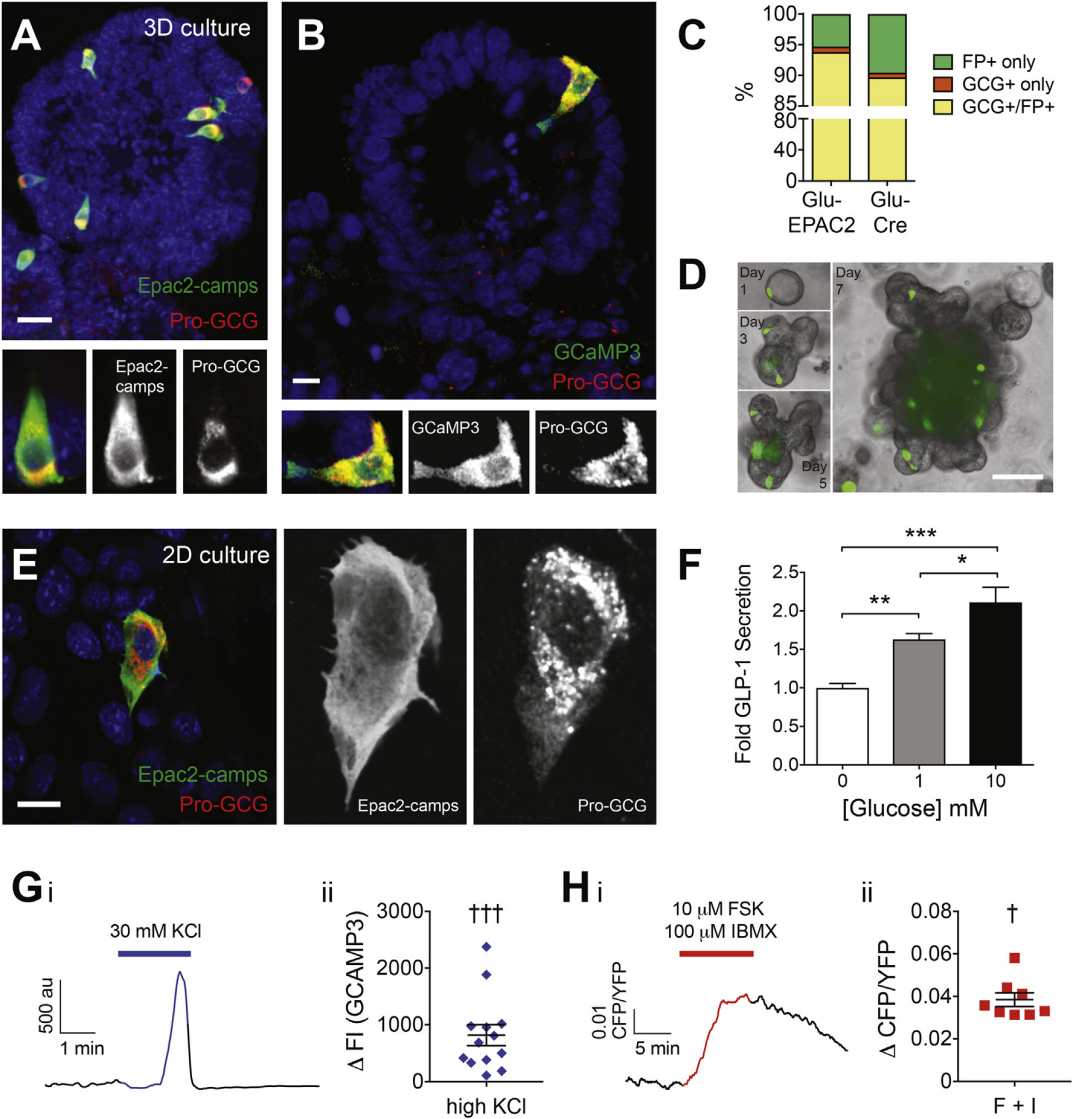
Characterization of *Glu-Cre* and *Glu-Epac2camps* ileal organoid lines. Projection of a confocal stack of an ileal-derived organoid from a *Glu-Epac2camps* (A) or *Glu-Cre* × *GCaMP3* (B) mouse showing immunostaining for Epac2camps or GCaMP3 (green), proglucagon (red) and nuclei (blue). Bottom panels show a single confocal optical slice of an ileal L-cell expressing both proglucagon and Epac2camps or GCaMP3. (C) Ileal organoids from *Glu-Cre* and *Glu-Epac2camps* lines scored for expression of fluorescent reporter (FP) and glucagon expression (GCG). (D) Organoid development (cyst to mature organoid) in the ileal *Glu-Epac2camps* line, showing both brightfield and Epac2camps fluorescence (green). (E) 2D organoid-derived cultures from ileal *Glu-Epac2camps* line immunostained for Epac2camps (green) and proglucagon (red). (F) Mean ± SEM (n = 9) fold GLP-1 secretion in 2D ileal organoid cultures in the presence of 1 or 10 mM glucose (gray or black bar, respectively) compared to buffer with no glucose (white bar). (G–H) Changes in intracellular calcium and cAMP in 2D ileal organoid L-cells using the *Glu-Cre* × *GCaMP3* and *Glu-Epac2camps* lines, respectively. (Gi) GCaMP3 fluorescence intensity (FI) over time in an ileal organoid L-cell perfused with 30 mM KCl in 1 mM glucose (blue line) or 1 mM glucose (black line) alone. (Gii) Scatterplot of GCaMP3 FI changes by 30 mM KCl (n = 13 cells), mean ± SEM also shown. (Hi) CFP/YFP ratio over time in an ileal organoid L-cell perfused with 1 mM glucose in the absence (black line) or presence of 10 μM Forskolin and 100 μM IBMX (red line). (Hii) Scatterplot of CFP/YFP ratio changes in response to addition of 10 μM Forskolin and 100 μM IBMX (n = 8 cells), mean ± SEM also shown. Scale bars A, B, and E = 10 μm, D = 500 μm. Statistical analysis performed using either a one-way ANOVA with Tukey's multiple comparison (F) or one sample t-test (G–H), p < 0.05 = */†, p < 0.01 = **, p < 0.001 = ***/†††.

Ileal organoids (each line derived from a single mouse with 2 independent lines for each genotype) were generated from mice expressing the FRET-based cAMP sensor Epac2camps or the fluorescent calcium sensor GCaMP3 under the control of the proglucagon promoter [4], [8], [9]. Immunofluorescent labeling confirmed that expression of both sensors was specific to cells producing proglucagon (Figure 1A–C), as previously reported for tissue sections from these mouse lines [4], [9]. The morphology of the developing organoids was similar to that described previously [12]: cysts developed multiple crypt domains, and fluorescently labeled L-cells were located in both the crypt and villus domains (Figure 1D, Supplemental Movie 1). Organoid lines were passaged every 7 days and maintained for more than 6 months and have been cryogenically frozen and re-established multiple times.

Supplementary video related to this article can be found at https://doi.org/10.1016/j.molmet.2017.11.005.

The following is the supplementary video related to this article:

Supplemental Movie 1

Well-established organoids containing multiple budding domains were dissociated into small cell clusters and plated onto dishes containing a thin coat of Matrigel (Figure 1E) to produce 2-dimensional (2D) cultures that brought L-cells into a single focal plane for imaging experiments, to allow free access for patch clamp electrodes, and to facilitate solution exchanges during live-cell experiments. Similar to both primary cultures from freshly-isolated intestinal crypts and intact 3D duodenal organoids [7], [12], [13], 2D ileal organoid cultures displayed GLP-1 secretory responses to elevated glucose concentrations, indicating that their constituent L-cells were functionally viable (Figure 1F).

In 2D cultures from Glu-Cre × GCaMP3 organoids, 30 mM KCl triggered a transient increase in GCaMP3 fluorescence consistent with the opening of voltage-gated Ca^2+^ channels and Ca^2+^ influx (Figure 1G). In Glu-Epac2camps organoids, the combination of forskolin (10 μM) and IBMX (100 μM) resulted in a robust increase in the CFP/YFP ratio, consistent with elevation of intracellular cAMP concentrations (Figure 1H). These results thus validate the GCaMP3 and Epac2camps reporter organoid lines for monitoring intracellular Ca^2+^ and cAMP changes in L-cells, and all further experiments were performed in 2D cultures.

### 3.2 Electrical activity of ileal L-cells in primary culture

**Figure 2:**
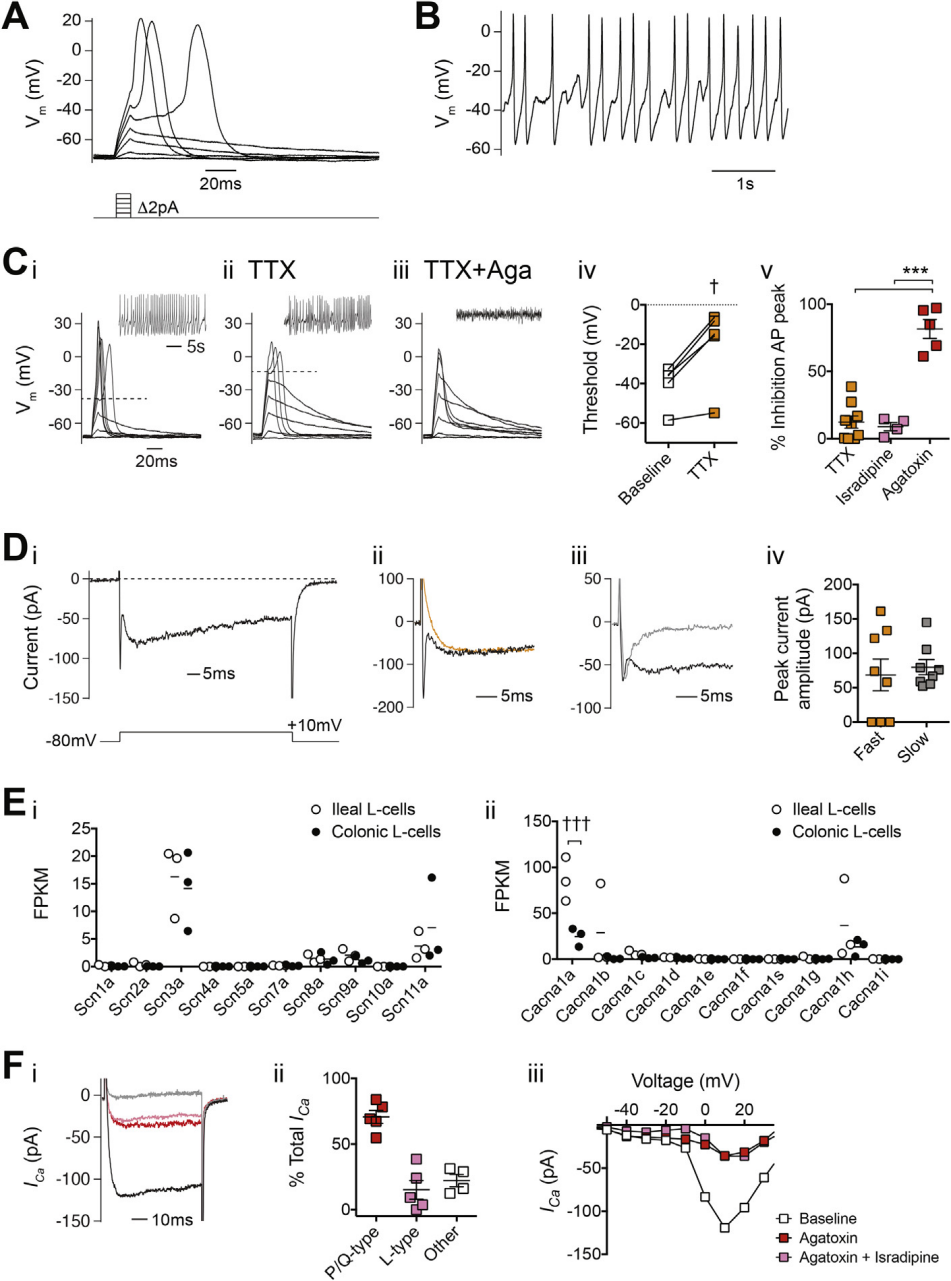
Electrophysiological characterization of organoid-derived ileal L-cells. (A) Perforated-patch current clamp recording of an organoid-derived ileal L-cell, firing action potentials in response to depolarizing current injections. Current was injected to maintain the cell at −70 mV, and a series of 10 ms current pulses were applied, increasing in magnitude by 2 pA. The pulse protocol is illustrated below. (B) Perforated-patch current clamp recording of spontaneous action potential firing from an ileal L-cell. (C) Representative traces using the same protocol as in (A), before (Ci) and during application of 0.3 μM tetrodotoxin (TTX, Cii) and during application of TTX + 0.5 μM ω-agatoxin-IVA (Ciii). Dashed line represents the threshold of action potential firing. The insets show spontaneous action potential firing under the same treatment conditions. (Civ) Threshold for action potential firing (n = 5) and (Cv) % inhibition of action potential peak following application of channel blockers, expressed as a % of total block by application of TTX (0.3 μM) + Cd^2+^ (100 μM). (Di) Inward current from a perforated-patch voltage clamp recording and sample traces following application of 0.3 μM TTX (Dii, orange trace) or 100 μM Cd^2+^ (Diii, gray trace). Currents were elicited from a series of 70 ms voltage steps from −110 to +60 mV, from a holding potential of −80 mV. Only the current response to the +10 mV voltage step is shown and is illustrated below the current traces. (Div) Peak current amplitude of the fast and slow current components. Gene expression data of *Scn* (Ei) or *Cacna* (Eii) genes by RNA sequencing of FACS-sorted L-cells from mouse ileum (white circles) and colon (black circles). Individual data points represent fragments per kilobase of transcript per million mapped reads (FPKM) from 1 mouse. Mean values (n = 3) are presented as lines. (Fi) Superimposed Ca^2+^ currents from an ileal L-cell before and during exposure to Ca^2+^ channel blockers. Red trace represents calcium current (*I*_Ca_) recorded in the presence of ω-agatoxin-IVA (0.5 μM), pink trace represents *I*_Ca_ recorded following subsequent application of isradipine (10 μM), and gray trace represents *I*_Ca_ recorded following application of cadmium (Cd^2+^, 100 μM). Currents were elicited using the protocol described in (D) and only the current responses to the +10 mV voltage step are shown. (Fii) Contribution of Ca^2+^ channel subtype to total Ca^2+^ current measured. (Fiii) The peak *I*_Ca_–voltage relationship for a representative organoid ileal L-cell following application of Ca^2+^ channel blockers. Statistical analysis performed using either by Wilcoxon matched-pairs signed rank test (Civ), one-way ANOVA with Tukey's multiple comparison (Cv) or multiple t-tests with Holm-Sidak multiple comparisons correction (E), p < 0.05 = †, p < 0.001 = ***/†††. Unless otherwise stated, each cell is represented as an individual point and lines on the graph represent mean ± SEM.

Electrophysiological recordings from ileal organoid-derived L-cells were performed in the perforated-patch configuration to preserve cytoplasmic signaling pathways, revealing that these cells are electrically excitable (Figure 2A and B). Action potentials triggered by short depolarizing current injections (Figure 2A) had a mean threshold of −29 ± 2 mV, reached peak potentials of +18 ± 2 mV, and had mean action potential half-widths of 29 ± 3 ms (n = 21). The majority of ileal organoid-derived L-cells (24/28 cells) fired spontaneous action potentials (Figure 2B; range of action potential firing frequency 0.3–3.5 Hz).

Compared with action potentials from colonic L-cells in primary short-term culture [18], the waveform of ileal organoid-derived L-cell action potentials was broader and had a lower peak, suggesting the involvement of a different profile of voltage-gated currents in ileal compared with colonic L-cells. To confirm that this difference was not due to the generation of ileal L-cells in organoid cultures, electrophysiological recordings were also obtained from ileal L-cells in primary culture, which are technically more demanding than colonic primary culture electrophysiological recordings. Action potential morphology was comparable in organoid-derived and primary ileal L-cells (Figure S1).

The Na^+^ channel blocker tetrodotoxin (TTX) had little effect on the action potential peak of ileal organoid L-cells (Figure 2Cii) but increased the action potential threshold (Figure 2Civ). By contrast, the action potential peak in either the presence or absence of TTX was substantially impaired by the selective P/Q-type Ca^2+^ channel blocker ω–agatoxin-IVA (0.5 μM, Figure 2Ciii and Cv) or the broad-spectrum Ca^2+^ channel blocker cadmium (Cd^2+^, 100 μM; Figure S1Aiii). These findings suggest that voltage-gated Na^+^ currents contribute to action potential initiation, but that peak potentials are predominantly determined by voltage-gated Ca^2+^ currents. Application of a selective L-type Ca^2+^ channel blocker isradipine (10 μM) had little effect on the action potential peak (Figure 2Cv). Voltage-clamp recordings revealed the presence of two types of inward current (Figure 2D): a fast component that was present in 5/8 cells and ablated by TTX (Figure 2Dii), and a slow current that was present in all cells and blocked by Cd^2+^ (Figure 2Diii). The characteristics of these fast and slow currents are typical for voltage-gated Na^+^ and Ca^2+^ currents, respectively.

We examined our RNA sequencing databases to determine the relative expression of voltage-gated Na^+^ and Ca^2+^ channel subunits in FACS-purified ileal and colonic L-cells (Figure 2E). Expression levels of the Na^+^ channel subunits *Scn3a* and *Scn11a* were comparable in ileal and colonic L-cells. The P/Q-type Ca^2+^ channel *Cacna1a* dominated the Ca^2+^ channel subunit profile and was more highly expressed in ileal than colonic L-cells. L-type (*Cacna1c*) and T-type (*Cacna1h*) subunits were also detected but at lower levels. Expression data for auxiliary subunits of voltage-gated Na^+^ and Ca^2+^ channels are shown in Figure S2A–B. Consistent with these expression profiles, ω–agatoxin-IVA blocked the majority of isolated Ca^2+^ currents in voltage-clamp recordings, whereas isradipine had a smaller effect (Figure 2F). A small component of non-L and P/Q-type current was suggested by the blockade of the remaining current by Cd^2+^.

### 3.3 Signaling pathways downstream of GPBAR1 in ileal organoid cultures

**Figure 3:**
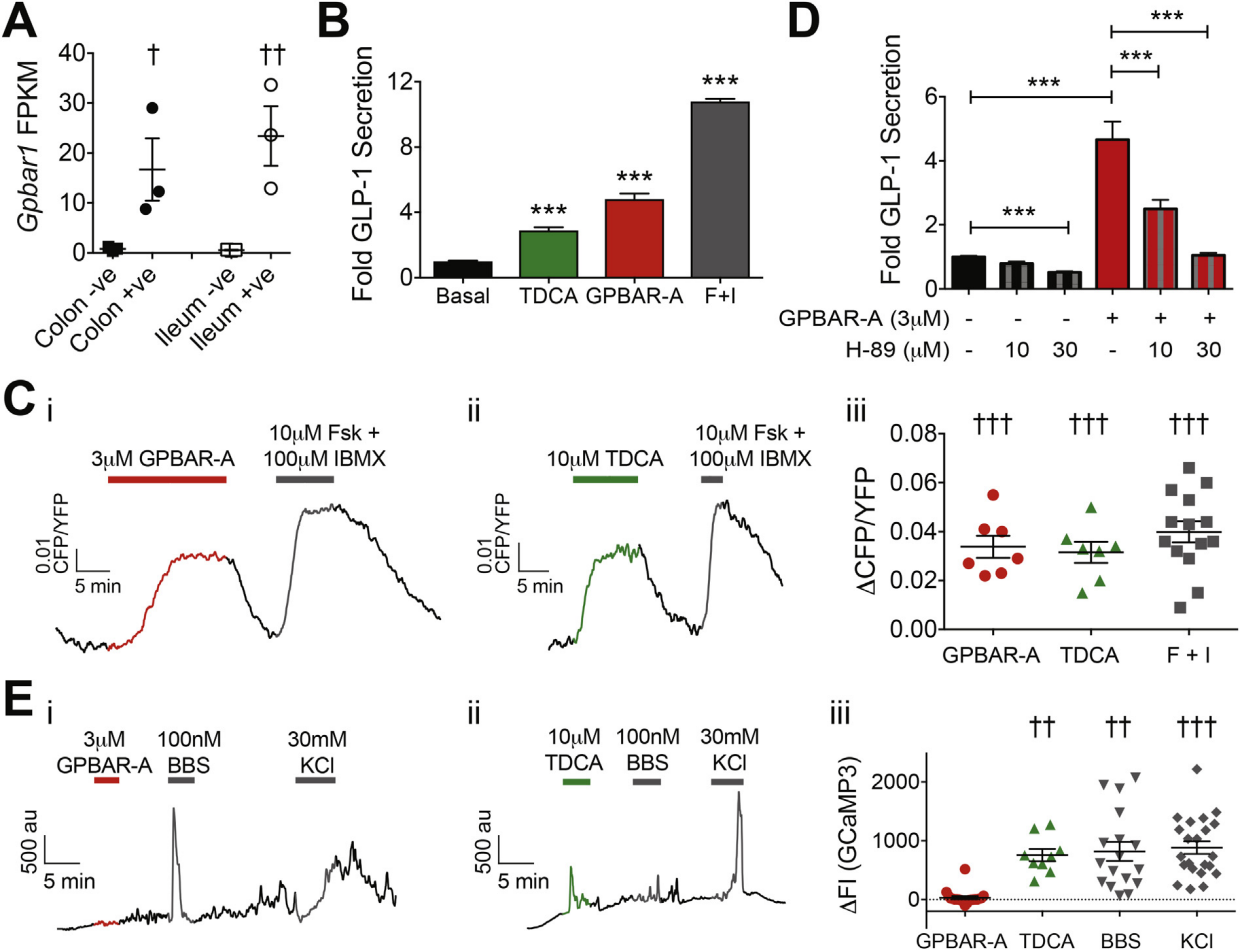
*Gpbar1* expression and responses to GPBAR1 agonists in 2D ileal organoid-derived L-cells. (A) Expression of *Gpbar1* by RNA sequencing of FACS-sorted L-cells (circles) and non-fluorescent control cells (squares) from mouse ileum (white) and colon (black). Individual data points represent sequencing results from 1 mouse and lines represent mean ± SEM (n = 3). (B) Mean ± SEM (n = 9) fold GLP-1 secretion in 2D ileal organoid cultures in the presence of 10 μM TDCA (green bar), 3 μM GPBAR-A (red bar), or 10 μM Forskolin +10 μM IBMX (gray bar) compared to 1 mM glucose (basal, black). (C) Changes in intracellular cAMP levels in 2D ileal organoids using the *Glu-Epac2camps* line. CFP/YFP ratio over time in response to 3 μM GPBAR-A (Ci, red line) or 10 μM TDCA (Cii, green line) treatment. 10 μM Forskolin + 100 μM IBMX (gray line) was used as a positive control stimulus. (Ciii) Scatterplot of ΔCFP/YFP ratio in ileal L-cells, in response to 3 μM GPBAR-A (n = 7), 10 μM TDCA (n = 7, 3 non-responders not plotted) and 10 μM Forskolin + 100 μM IBMX (F + I) (n = 14). Scatterplot shows individual cell responses with mean ± SEM also shown. (D) Mean ± SEM (n ≥ 9, data pooled from 5 independent experiments) GLP-1 secretion (fold compared to basal) in 2D ileal organoid cultures in the presence and absence of the PKA inhibitor H-89 (10 or 30 μM) and GPBAR-A (3 μM) compared to basal (1 mM glucose) as indicated. (E) Changes in intracellular Ca^2+^ in organoid-derived L-cells. Example traces of changes in GCaMP3 FI over time in response to 3 μM GPBAR-A (Ei, red line) or 10 μM TDCA (Eii, green line) treatment on top of 1 mM glucose, with 100 nM Bombesin (BBS) and/or 30 mM KCl used as a positive control stimulus (gray line). (Eiii) Scatterplot of changes in GCaMP3 FI in ileal L-cells, in response to 3 μM GPBAR-A (n = 11), 10 μM TDCA (n = 11), 100 nM Bombesin (n = 12) and 30 mM KCl (n = 14). Scatterplot shows individual cell responses with mean ± SEM also shown. Statistical analysis performed using either paired t-test (A), one-way ANOVA with Dunnett's multiple comparison (B), a one sample t-test (C and E), or one-way ANOVA with Bonferroni multiple comparisons correction (D), p < 0.05 = †, p < 0.01 = ††, p < 0.001 = ***/†††.

2D ileal organoid-derived cultures were treated with agonists targeting GPBAR1, which is highly expressed in ileal L-cells (Figure 3A) [7]. GLP-1 secretion was enhanced ∼3-fold by the bile acid taurodeoxycholic acid (TDCA), 5-fold by a small molecule GPBAR1 agonist GPBAR-A [19], and >10-fold by forskolin/IBMX (Figure 3B). These results are similar to those we reported previously for primary short-term ileal cultures [15].

As predicted from the previously reported coupling of GPBAR1 to G_αs_ [6], TDCA or GPBAR-A robustly elevated L-cell cAMP concentrations (Figure 3C), and, in secretion studies, GPBAR-A-triggered GLP-1 release was blocked by PKA inhibition (Figure 3D). L-cell Ca^2+^ responses were triggered by TDCA but not GPBAR-A (Figure 3E), as also previously observed in primary cultures [15], likely reflecting GPBAR1-independent TDCA effects. Although GPBAR1 has been reported previously to couple to TRPA1 [20], a channel expressed highly in L-cells [8], GPBAR-A-triggered GLP-1 secretion was not impaired by TRPA1 inhibition (Figure S3A).

**Figure 4:**
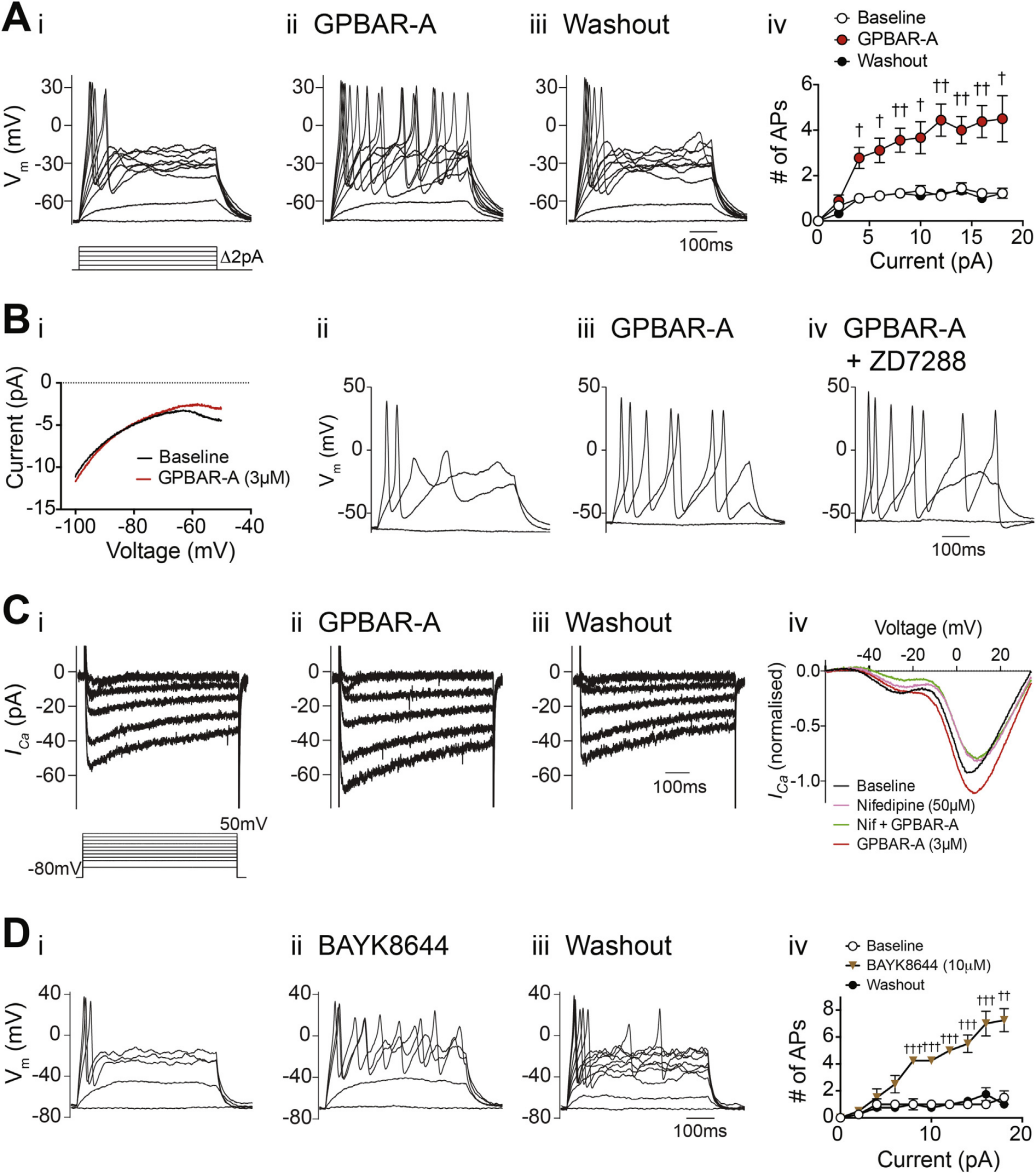
Electrophysiological responses of organoid-derived ileal L-cells to GPBAR1 agonists. (A) Perforated-patch current clamp recording of an L-cell firing action potentials evoked by depolarizing current injections before (i), during (ii) and after (iii) application of GPBAR-A (3 μM). Current was injected to maintain the cell at −70 mV, and a series of 500 ms current pulses was applied, increasing in magnitude by 2 pA. (Aiv) Mean number (n = 9) of action potentials (threshold −10 mV) elicited during current injections as in Ai-iii, with error bars representing SEM. (Bi) Current–voltage relationship as assessed by voltage ramps over 500 ms from −100 to −50 mV, from a holding potential of −80 mV. Twenty ramps per condition in each cell (n = 11 cells) were averaged to represent baseline (black trace) and GPBAR-A treatment (3 μM, red trace). Recording of an L-cell studied by the same protocol as (A), before (Bii), during application of GPBAR-A (3 μM, Biii), and additional application of the HCN channel blocker, ZD7288 (10 μM, Biv). Perforated-patch voltage clamp recordings of *I*_Ca_ before (Ci), during (Cii) and after (Ciii) application of GPBAR-A (3 μM). (Civ) *I*_Ca_–voltage relationship of ileal L-cells as assessed with 160 ms voltage ramps from a holding potential of −80 mV to +80 mV, during application of GPBAR-A (3 μM) and nifedipine (50 μM). Ten voltage ramps were averaged per treatment for each cell (n = 6) and normalized to the baseline peak *I*_Ca_ amplitude. Recording of an ileal L-cell, elicited by the same protocol as (A) before (Di), during (Dii) and after (Diii) application of BayK8644 (10 μM). (Div) Mean number of action potentials (threshold −10 mV) elicited during current injections as in Di-iii, with error bars representing SEM (n = 4). Statistical significance was assessed using multiple t-tests with Holm-Sidak multiple comparisons correction (Aiv and Div), p < 0.05 = †, p < 0.01 = ††, p < 0.001 = †††.

In perforated patch recordings, GPBAR-A did not alter the morphology of evoked action potentials or affect the inter-spike membrane potential (Figure S3B), but triggered a reversible increase in action potential frequency during prolonged depolarizing current injections (Figure 4A) that was further enhanced at larger current injections (Figure 4Aiv). Ramp current–voltage relationships between −100 and −50 mV did not reveal any measurable conductance changes (Figure 4Bi), suggesting that the increased firing frequency was not due to a reduction in background potassium current or increased voltage-independent inward current. We also did not observe any recruitment of hyperpolarisation activated (HCN) currents by GPBAR-A (Figure S3C), and the effect of GPBAR-A on evoked action potential frequency was unaffected by the HCN-antagonist ZD7288 (10 μM; Figure 4Biv). We further examined the role of voltage-gated Ca^2+^ channels, and found that GPBAR-A increased the peak Ca^2+^ current amplitude by 20.2 ± 3.4% (n = 12, Figure 4C). This effect of GPBAR-A was not observed in the presence of the L-type Ca^2+^ channel blocker nifedipine (50 μM), suggesting that GPBAR1 activation enhances the activity of L-type Ca^2+^ channels, consistent with the known modulation of these ion channels by PKA [21]. We attempted to address whether GPBAR1-dependent activation of L-type Ca^2+^ channels was responsible for the increased action potential frequency during prolonged depolarizing pulses, using the inhibitor nifedipine (50 μM) and verapamil (10 μM). Unfortunately, we observed a sustained depolarization using this protocol in the presence of L-type channel blockers, possibly reflecting previously-reported inhibitory effects of these drugs on voltage-gated potassium channels [22](Figure S4). Evoked action potential firing was increased by the L-type Ca^2+^ channel activator BayK8644 (10 μM; Figure 4D), supporting the hypothesis that enhanced L-type Ca^2+^ channel activity could contribute to the increased action potential firing observed in the presence of GPBAR-A, although off-target effects of this dihydropyridine-derivative on voltage-gated potassium channels have also been reported [23].

### 3.4 Signaling pathways downstream of FFA1 in ileal organoid cultures

**Figure 5:**
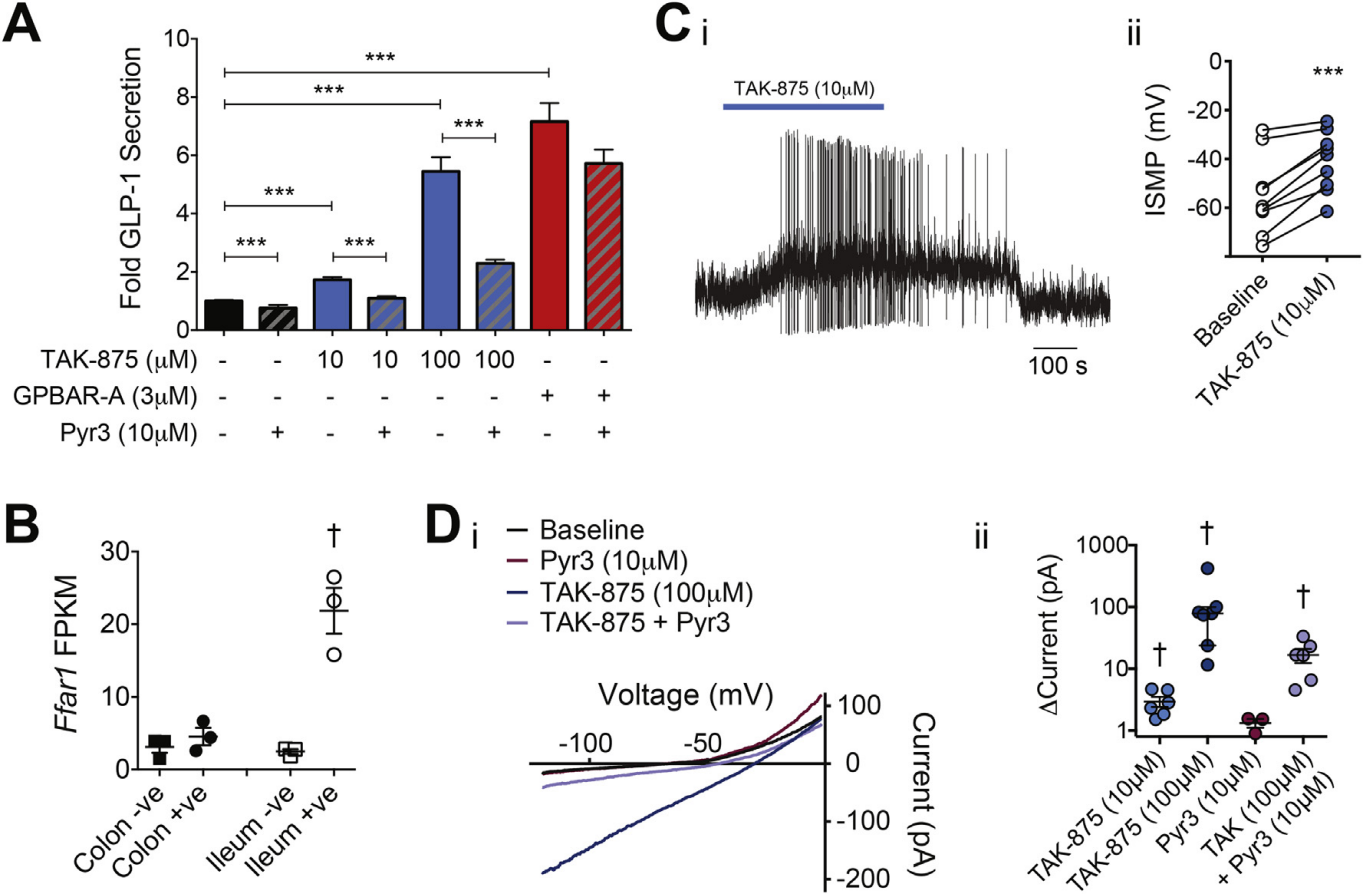
FFA1 stimulation recruits TRPC3 in organoid-derived ileal L-cells. (A) Mean ± SEM (n = 9) fold GLP-1 secretion in the presence or absence of TAK-875 (10 or 100 μM, blue bars), Pyr3 (10 μM, striped bars) and GPBAR-A (3 μM, red bars) as indicated compared to basal (1 mM glucose, black bars). (B) Expression of *Ffar1* by RNA sequencing in FACS-sorted L-cells (circles) and non-fluorescent control cells (squares) from mouse ileum (white) and colon (black). Individual data points represent sequencing results from 1 mouse, mean ± SEM (n = 3) also shown. (Ci) Perforated-patch current clamp recording of an L-cell during application of TAK-875 (10 μM). (Cii) Change (n = 8) in measured inter-spike membrane potential (ISMP) during application of TAK-875 (10 μM). (Di) Current–voltage relationship as assessed by voltage ramps over 120 ms from −120 to 0 mV, from a holding potential of −70 mV, and (Dii) Scatterplot of changes in current (Δcurrent) in response to 10 μM TAK-875 (n = 6), 100 μM TAK-875 (n = 7), 10 μM Pyr3 (n = 3), and 100 μM TAK-875 + 10 μM Pyr3 (n = 6). Median ± interquartile range shown. Statistical analysis performed using either a one-way ANOVA with Bonferroni multiple comparisons correction (A), a paired t-test (B and Cii), or Wilcoxon signed rank test (Dii), p < 0.05 = †, p < 0.001 = ***.

The FFA1 agonist TAK-875 triggered a small increase in GLP-1 secretion at 10 μM and a more robust secretory response at 100 μM, in 2D ileal organoid cultures (Figure 5A), consistent with the expression of *Ffar1* in FACS-purified ileal L-cells by RNAseq analysis (Figure 5B). In electrophysiological current clamp recordings, 10 μM TAK-875 triggered L-cell membrane depolarization by a mean of 14 ± 2 mV, which in 4/9 cells was sufficient to trigger or increase action potential firing (Figure 5C). In voltage clamp recordings, 10 and 100 μM TAK-875 activated an inward current that was blocked by an inhibitor of TRPC3 channels, Pyr3 (10 μM; Figure 5D). Furthermore, Pyr3 reduced GLP-1 secretion triggered by TAK-875 (Figure 5A), supporting the role of TRPC3 channels downstream of FFA1 activation in mediating GLP-1 release. TAK-875 (10 μM) additionally reduced the peak voltage-gated Ca^2+^ current amplitude by 10.9 ± 1.6% (n = 6, Figure S5), consistent with the reported inhibition of P/Q-type currents by Gq coupled receptors [24].

### 3.5 Interactions between signaling pathways activated by GPBAR1 and FFA1

**Figure 6:**
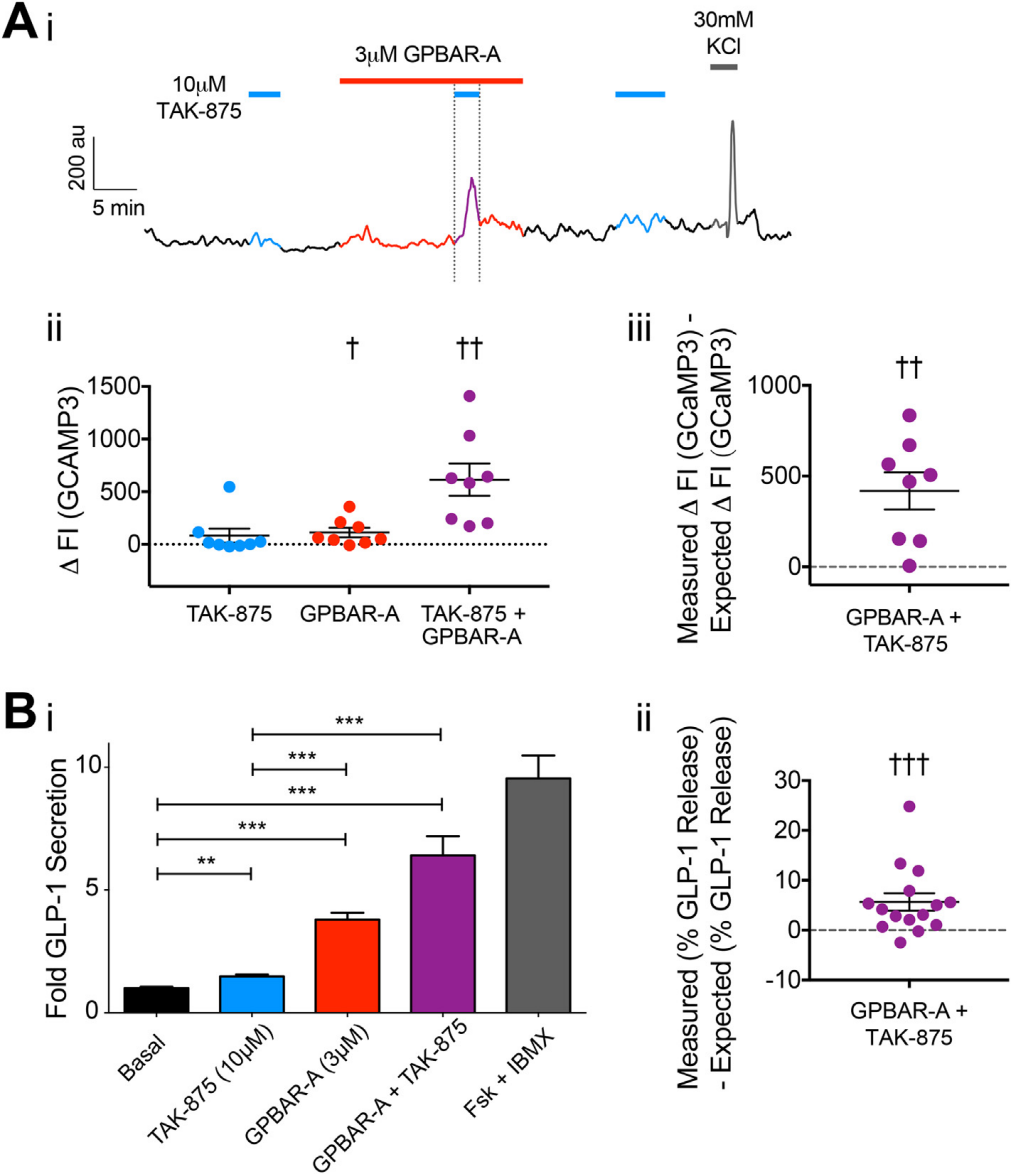
Synergistic effects of FFA1 and GPBAR1 on intracellular calcium levels and GLP-1 secretion. (A) Changes in intracellular Ca^2+^ levels in organoid-derived L-cells using the *Glu-Cre* × *GCaMP3* line. (Ai) Changes in GCaMP3 FI over time in response to 10 μM TAK-875 (blue line), 3 μM GPBAR-A (red line), co-application of 10 μM TAK-875 and 3 μM GPBAR-A (purple line) on top of 1 mM glucose, with 30 mM KCl used as a positive control stimulus (gray line). (Aii) Scatterplot of changes in GCaMP3 FI in L-cells, in response to 10 μM TAK-875, 3 μM GPBAR-A, and co-application of 10 μM TAK-875 and 3 μM GPBAR-A. Scatterplot shows individual cell responses with median ± interquartile range (n = 8) shown in black. (Aiii) Measured − expected change in GCaMP3 FI in response to co-application of GPBAR-A and TAK-875 for the cells shown in Aii. (Bi) Mean ± SEM (n = 15) fold GLP-1 secretion during single applications of 10 μM TAK-875 (blue bar), 3 μM GPBAR-A (red bar), and co-application of both drugs (purple bar) compared to basal (1 mM glucose alone), 10 μM Forskolin + 10 μM IBMX (gray bar) treatment used as a positive control. (Bii) Measured − expected change in % GLP-1 secretion upon co-application of GPBAR-A and TAK-875. The gray dashed line (at 0) represents no synergy. Statistical analysis performed using either Wilcoxon signed-rank test (Aii, Bii), one-way ANOVA with Bonferroni's multiple comparison (Bi) or one-sample t-test (Aiii), p < 0.05 = †, p < 0.01 = **/††, p < 0.001 = ***/†††.

The lower TAK-875 concentration of 10 μM was selected to investigate potential synergy with the GPBAR1 pathway. At this concentration, TAK-875 on its own was a poor stimulus of Ca^2+^ responses in L-cells as monitored by GCaMP3 (Figure 6A), and triggered only a small elevation of GLP-1 secretion (Figure 6Bi). In the presence of GPBAR-A, co-application of TAK-875 triggered significantly larger Ca^2+^ responses, which in 7/8 cells analyzed were greater than the additive effects of GPBAR-A and TAK-875 administered singly (Figure 6Aii–iii). The combination of GPBAR-A with TAK-875 also evoked a synergistic increase in GLP-1 secretion that was evident in 12/15 wells tested across 5 experiments (Figure 6Bii). In contrast, combination of BayK8644 (1 μM) and TAK-875 (10 μM) did not result in a greater than additive stimulation of GLP-1 secretion (Figure S4C).

## 4 Discussion

For this study, we established and characterized ileal organoid lines from reporter mice expressing biosensors for intracellular calcium and cAMP levels in proglucagon expressing L-cells. To utilize the model for multiple assay platforms, we developed a 2D culture method that recapitulated GLP-1 secretory responses to known stimuli such as glucose and agonists of GPBAR1 and FFA1 [4], [6], [7], [13], [15], [25], [26]. One of the main advantages of the 2D organoid-derived cultures was the improved accessibility of cells to patch-clamp electrodes, which enabled the electrophysiological characterization of ileal L-cells.

The electrical properties of ileal L-cells were not identical to those previously described in GLUTag and colonic L-cells [18], [27], likely reflecting the observed regional differences in expression profiles of voltage-gated Na^+^ and Ca^2+^ channel subunits. Voltage-gated Na^+^ channels appeared important for action potential initiation in ileal L-cells, as the threshold for action potential firing was increased to ∼−15 mV in the presence of TTX. This new threshold matched the potential at which voltage-gated Ca^2+^ currents were observed to activate, consistent with the finding that the P/Q-type Ca^2+^ channel blocker ω-agatoxin-IVA significantly lowered the action potential peak height (Figure 2Ciii). In colonic L-cells, by contrast, we previously reported that voltage-gated Na^+^ currents were larger and contributed significantly to the action potential peak. In both ileal and colonic L-cells; however, we observed that Ca^2+^-dependent action potential firing was possible even in the presence of TTX. Differences between Ca^2+^ currents in ileal vs colonic L-cells could possibly arise from altered expression of auxiliary subunits such as α2δ1 subunits (*Cacna2d1*), which play a role in channel assembly and trafficking [28] and were more abundant in ileal than colonic L-cells (Figure S2Aii).

*Gpbar1* and *Ffar1* were both highly expressed in primary ileal L-cells (Figure 3, Figure 5B), consistent with the measured increases in GLP-1 secretion following activation of either receptor in ileal organoid-derived cultures (Figure 3, Figure 5A). GPBAR-A elevated cAMP levels and increased evoked action potentials in ileal L-cells. Although cAMP elevation was linked to activation of HCN currents in GLUTag cells [29], we did not observe any effect of GPBAR-A on hyperpolarization-activated or voltage-independent currents in ileal L-cells. GPBAR-1 did, however, increase the activity of L-type Ca^2+^ currents by ∼20%, consistent with previous observations of PKA-dependent regulation of these channels in other excitable cells [21]. GPBAR1 has been reported to couple to TRPA1 [20], but we observed no effect of TRPA1-antagonism on GPBAR-A-triggered GLP-1 secretion (Figure S3A), and in electrophysiological recordings we saw no evidence for the appearance of TRPA1-currents after GPBAR1 activation. This suggests that TRPA1 activity was low under our culture conditions. Future work will address the role and recruitability of TRPA1 in L-cells, as its activation would nonetheless increase the excitability of L-cells by driving them closer towards the threshold of voltage-gated Ca^2+^-channel activation.

TAK-875 triggered L-cell membrane depolarization by activating an inward current that was blocked by the TRPC3 inhibitor Pyr3, supporting previous data suggesting a role for this channel downstream of FFA1 in L-cells and pancreatic β-cells [30], [31]. The TAK-875 triggered currents were ∼80% reduced by Pyr3, either reflecting incomplete inhibition of TRPC3 by this drug [32] or indicating a small contribution of additional conductances. TRPC3 is a non-selective Na^+^/Ca^2+^ channel [33], activation of which in β-cells was abolished by inhibitors of PLC or PKC [30]. Despite the depolarizing effect of 10 μM TAK-875 on the L-cell membrane potential, only small L-cell Ca^2+^ and GLP-1 secretory responses were observed at this drug concentration, and action potential firing was only triggered in a proportion of L-cells. The small Ca^2+^ response might in part be due to the low glucose concentration used in these experiments (1 mM), as increased insulin secretion from pancreatic β-cells by TAK-875 was highly dependent upon the glucose concentration [30]. At low glucose, the membrane depolarization triggered by 10 μM TAK-875 might be insufficient to open enough voltage-gated Ca^2+^ channels to activate secretion in the majority of L-cells. Higher TAK-875 concentrations triggered robust GLP-1 secretory responses, consistent with the activation of a large depolarizing TRPC3 current.

Simultaneous activation of FFA1 and GPBAR1 synergistically stimulated GLP-1 secretion consistent with previous observations [6], although the underlying mechanism for the interaction was previously unknown. Ca^2+^ responses to co-application of GPBAR-A with TAK-875 (Figure 6A) were also significantly greater than the sum of the responses to the agents added individually, suggesting that synergy between the signaling pathways occurs, at least in part, before or at the level of Ca^2+^ entry. We hypothesize that the synergy between GPBAR1 and FFA1 on Ca^2+^ responses arises because the net effect of GPBAR1 and FFA1 activation is to increase the activity of voltage-gated Ca^2+^ channels (20% activation by GPBAR1 vs 11% inhibition by FFA1). At membrane potentials below the activation threshold for voltage-gated Ca^2+^ currents, this has little effect, but when the membrane is depolarized by FFA1-dependent TRPC3 channel opening, this triggers a larger Ca^2+^ response. The enhanced activity of L-type channels by GBAR1-dependent activation and membrane depolarization likely also contributes to the increase in hormone secretion.

## 5 Conclusions

2D cultures produced from murine ileal organoids are a good model system for studying L-cell function, recapitulating the properties of freshly cultured ileal epithelium as well as the intact perfused intestine. Our finding that GPBAR1 agonists enhanced the response to membrane depolarization triggered by FFA1 agonism is likely a general mechanism applicable to a variety of stimulus combinations. The action of GPBAR1 included PKA-dependent activation of L-type Ca^2+^ currents, which have been closely linked to vesicular exocytosis in pancreatic β-cells [34], and would likely be mimicked by other G_αs_-coupled receptors. This, in turn, should enhance the responsiveness to any depolarizing stimulus, including sodium coupled glucose uptake by SGLT1, proton coupled dipeptide uptake by PEPT1 and activation of other G_αq_-coupled GPCRs that recruit TRPC3 or alternative non-selective cation channels. While the electrophysiological integration of different signals described here seems sufficient to explain the observed synergistic Ca^2+^ response observed in the presence of TAK-875 plus GPBAR-A, we were unable to simulate a synergistic effect on GLP-1 secretion by the combination of the L-type Ca^2+^-channel activator BayK8644 and TAK-875. Therefore, we believe that other cAMP targets such as Epac2 (which is highly expressed in L-cells) likely contribute to L-cell integration of different stimuli at the levels of GLP-1 secretion, e.g. affecting the pool of readily releasable vesicles, as has been reported previously in pancreatic β-cells [35].

Both GPBAR1 and FFA1 are under investigation as candidate drug targets for increasing gut hormone secretion in humans, and thereby treating conditions such as type 2 diabetes and obesity. Our data confirm and help to explain previous observations that drug combinations targeting G_αs_ as well as G_αq_ have greater efficacy on gut hormone secretion than single agents, and further raise the important idea that agents activating G_αs_-coupled receptors are likely to have synergistic actions with a number of dietary components including glucose, and therefore to exhibit differential effects on GLP-1 secretion dependent on concomitant food ingestion.

## Author contributions

DAG, VBL, LJB, PL, and GT performed the research. DAG, VBL, LJB, GT, FR, and FMG designed the research study. DAG, VBL, LJB, and PL analyzed the data. DAG, VBL, FR, and FMG wrote the paper. All authors edited the paper for intellectual content and approved its publication.

## Appendix A Supplementary data

mmc1
