# Supplementary material for: Mechanistic insights into the detection of free fatty and bile acids by ileal glucagon-like peptide-1 secreting cells
Source: Mol Metab. 2017 Nov 11;7:90–101. doi: 10.1016/j.molmet.2017.11.005 (PMC5784317; doi:10.1016/j.molmet.2017.11.005)
Supplement: mmc1 [file mmc1.pdf]

Supplemental Figures:

Supplemental Figure 1: Electrophysiological properties of primary ileal L-cells in comparison with organoid-derived ileal L-cells

(A) Perforated-patch current clamp recording of a primary ileal L-cell, displaying action potentials triggered by depolarising current injections. Current was injected to maintain the cell at −70 mV, and a series of 10 ms current pulses were applied, increasing in magnitude by 2 pA. The pulse protocol is illustrated below. Representative traces show action potential firing before (Ai) and during application of 0.3 µM tetrodotoxin (TTX, Aii), and during application of TTX + 100 µM Cd^{2+} (Aiii). Dashed line represents the threshold of action potential firing. The insets show spontaneous action potential firing at resting membrane potential (RMP) under the same treatment conditions. (B) Comparison of action potential properties between organoid-derived (white circles, n=21) and primary (grey squares, n=4) ileal L-cells from voltage protocol described in (A).

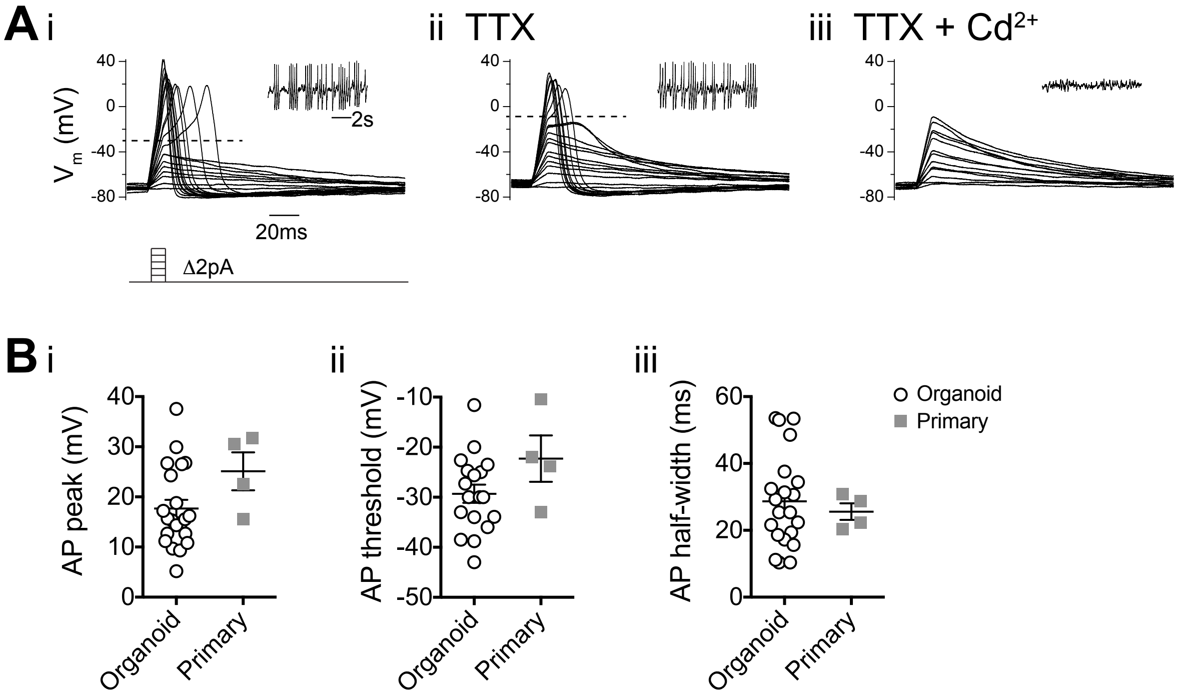

A i
ii TTX
iii TTX + Cd2+
Vm (mV)
40
0
-40
-80
2s
20ms
Δ2pA
B i
ii
iii
AP peak (mV)
AP threshold (mV)
AP half-width (ms)
Organoid
Primary

Supplemental Figure 2: Transcript levels of voltage-gated Ca^{2+} and Na^{+} channel accessory subunits

Gene expression data of voltage-gated Ca^{2+} channel accessory subunits (A), and voltage-gated Na^{+} channel accessory subunits (B) by RNA sequencing of FACS-sorted L-cells from mouse ileum (white circles) and colon (black circles). Individual data points represent sequencing results from 1 mouse (N = 3). Mean ± SEM are also presented as a line. Statistical analysis performed using multiple t-tests with Holm-Sidak multiple comparisons correction, $p<0.001$ = †††.

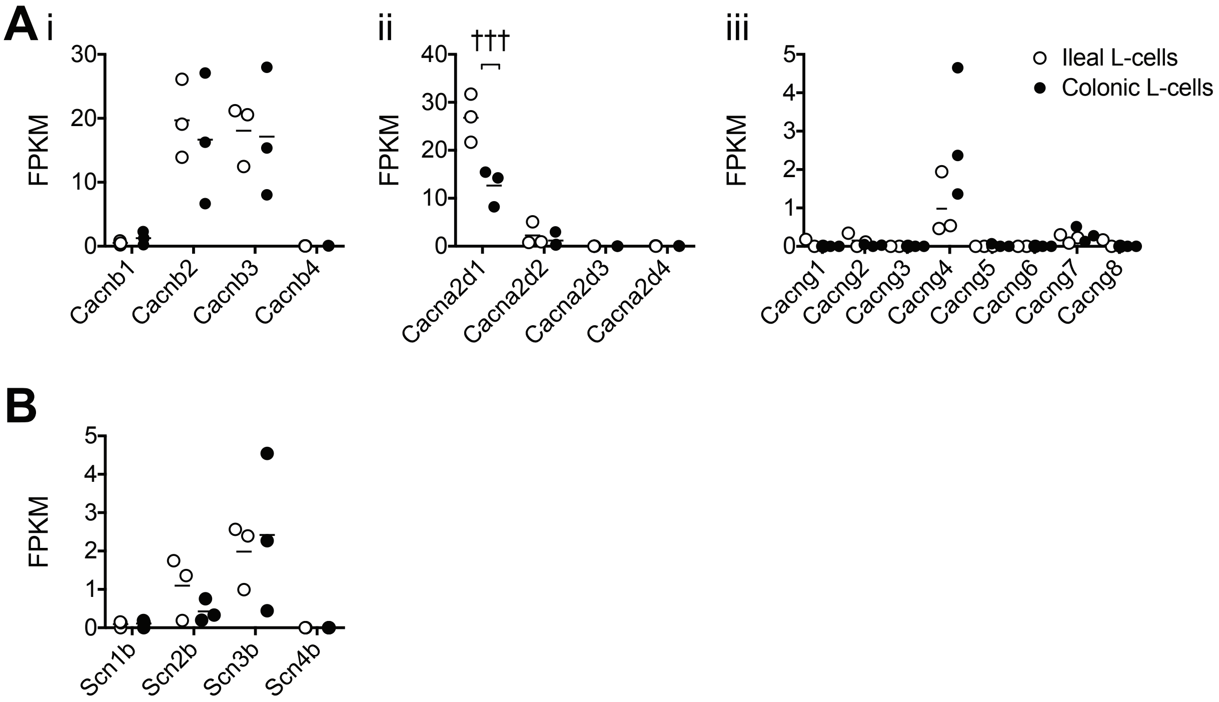
A
i
FPKM
30
20
10
0
Cacnb1
Cacnb2
Cacnb3
Cacnb4
ii
†††
40
30
20
10
0
Cacna2d1
Cacna2d2
Cacna2d3
Cacna2d4
iii
Ileal L-cells
Colonic L-cells
5
4
3
2
1
0
Cacng1
Cacng2
Cacng3
Cacng4
Cacng5
Cacng6
Cacng7
Cacng8
B
FPKM
5
4
3
2
1
0
Scn1b
Scn2b
Scn3b
Scn4b

Supplemental Figure 3: GPBAR1 activation does not involve TRPA1, membrane depolarisation or activation of HCN currents

(A) Plot showing median ± interquartile range (n=12) fold GLP-1 secretion in 2D ileal organoid cultures in the presence of 3 µM GPBAR-A, 50 µM Carvacrol and 10 µM Forskolin + 10 µM IBMX compared to 1 mM glucose alone. Also shown TRPA1 inhibition with 50 µM HC030031 or 10 µM A967079. All data points from each experiment are plotted. (B) Change in measured ISMP during application of GPBAR-A (3 µM). (C) Currents recorded during application of GPBARA (3 µM), elicited by a series of 2 s voltage steps from −50 to −140 mV applied from a holding potential of −50 mV.

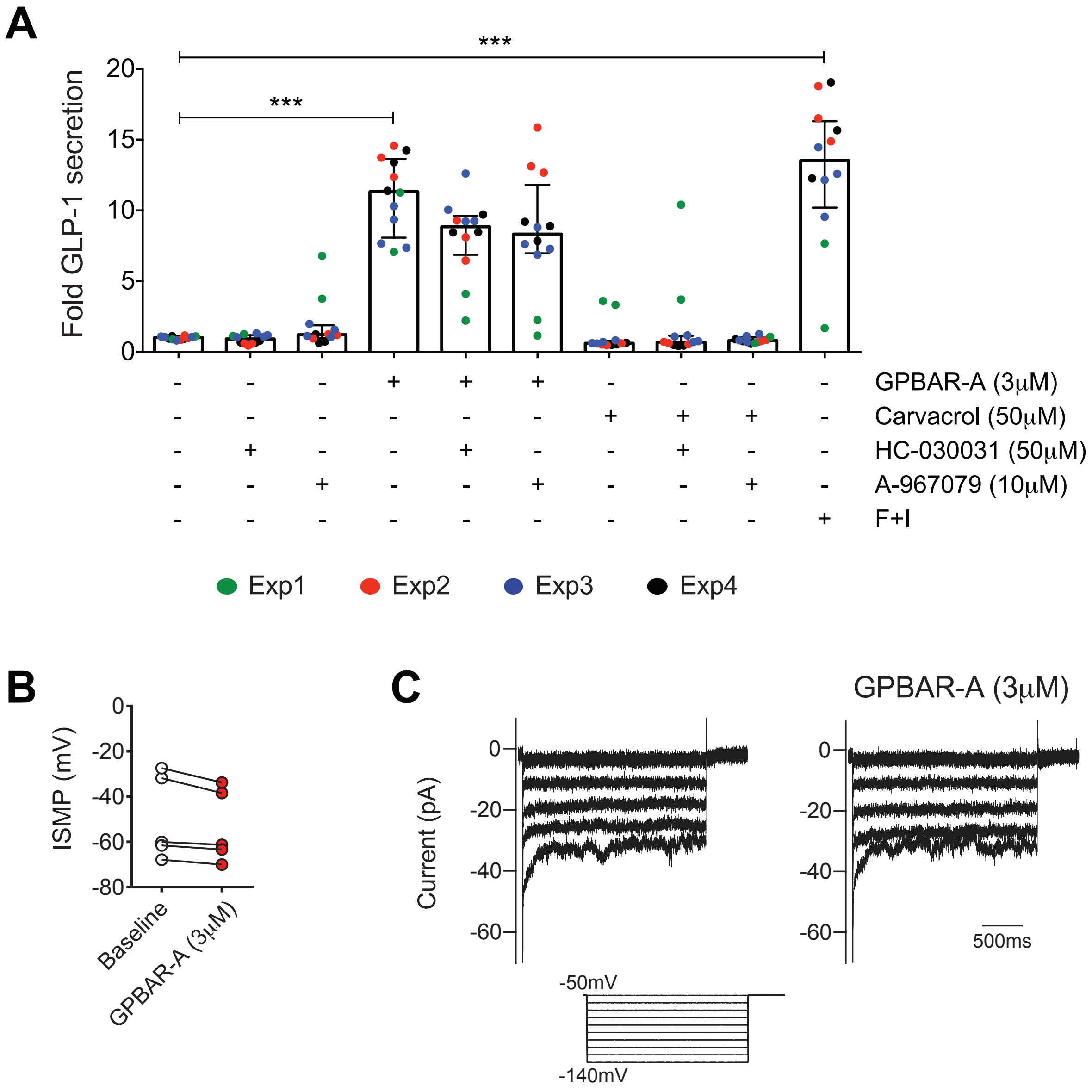

A
***
***
Fold GLP-1 secretion
20
15
10
5
0
GPBAR-A (3μM)
Carvacrol (50μM)
HC-030031 (50μM)
A-967079 (10μM)
F+I
Exp1
Exp2
Exp3
Exp4
B
ISMP (mV)
0
-20
-40
-60
-80
Baseline
GPBAR-A (3μM)
C
GPBAR-A (3μM)
Current (pA)
0
-20
-40
-60
500ms
-50mV
-140mV

Supplemental Figure 4: Effects of L-type Ca^{2+}-channel inhibitors on electrophysiological responses to prolonged depolarisation and the L-type channel activator BAYK8644 on GLP-1 secretion

(A) Perforated-patch current clamp recording from an organoid-derived ileal L-cell before and during application of 50 µM nifedipine. Current was injected to maintain the cell at –70 mV and responses were triggered by superimposed pulses of increasing magnitude (Δ4 pA). (B) Similar recording as (A) but during application of 10 µM verapamil. (C) Mean ± SEM (n=9) fold GLP-1 secretion in 2D ileal organoid cultures in response to TAK-875 (10 mM) and/or BAYK8644 (1 mM), as indicated. Data points collected in parallel on the same day are shown in the same colour. Statistical analysis performed using one-way ANOVA with a Dunnett's multiple comparisons test, $p<0.05$ = *, $p<0.001$ = ***.

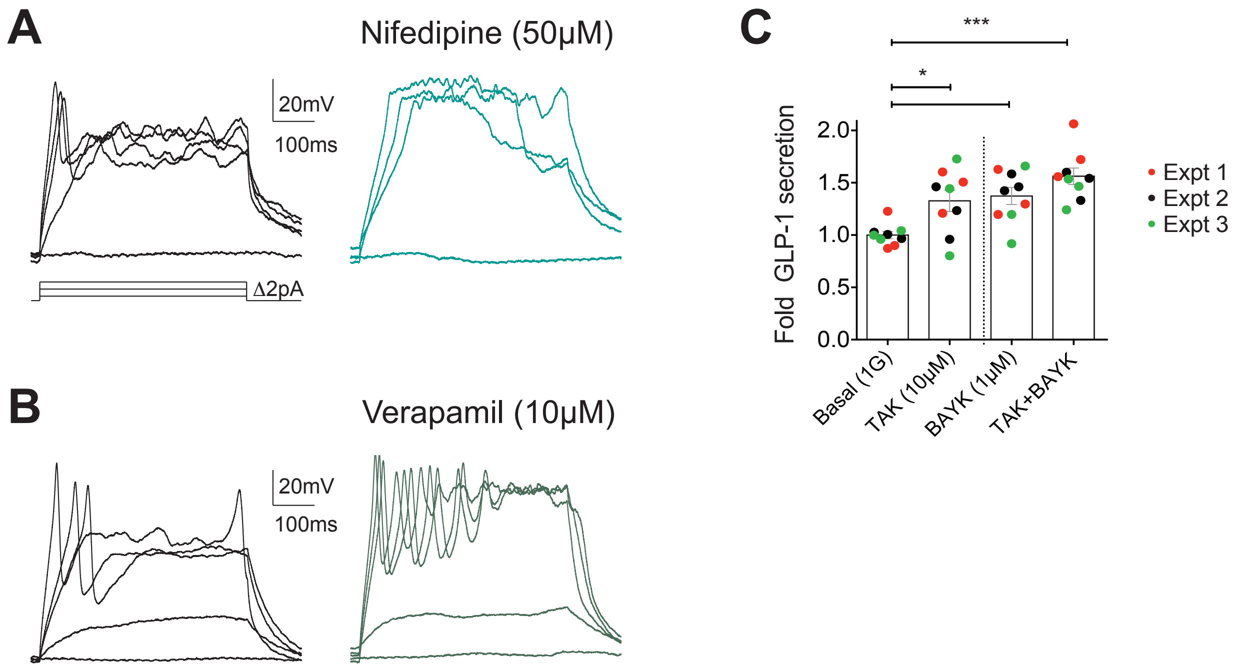

A
Nifedipine (50μM)
20mV
100ms
Δ2pA
B
Verapamil (10μM)
20mV
100ms
C
***
*
Fold GLP-1 secretion
2.0
1.5
1.0
0.5
0.0
Basal (1G)
TAK (10μM)
BAYK (1μM)
TAK+BAYK
Expt 1
Expt 2
Expt 3

Supplemental Figure 5: Effects of FFA1 activation by TAK-875 on L-type Ca^{2+}-currents

(A) Ca^{2+} current-voltage curves from a representative organoid-derived ileal L-cell in response to Pyr3 (10 µM, purple), Pyr3+TAK-875 (10 µM each, cyan), Pyr3+TAK-875 (10 & 100 µM, blue), washout (grey) and forskolin (10 µM, green), applied in that order. Currents were recorded in the perforated patch configuration using solutions designed to isolate Ca^{2+} currents. Ten voltage ramps, 160 ms from –80 to +80 mV from a holding potential of –80 mV, were averaged per treatment. (B) Scatterplot of % change in peak I_{Ca} (n=6). Individual cell responses per treatment shown with mean ± SEM also represented. Statistical analysis performed using one-way ANOVA with a Dunnett's multiple comparisons test, $p<0.05$ = *, $p<0.001$ = ***.

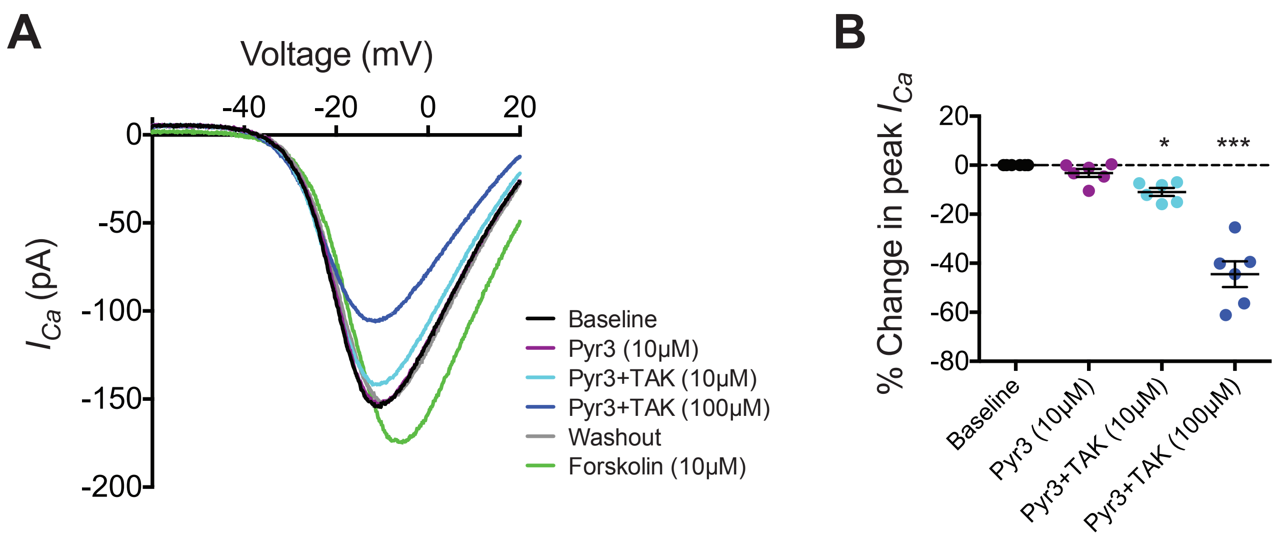
A
Voltage (mV)
-40
-20
0
20
0
-50
-100
-150
-200
I_{Ca} (pA)
Baseline
Pyr3 (10μM)
Pyr3+TAK (10μM)
Pyr3+TAK (100μM)
Washout
Forskolin (10μM)
B
% Change in peak I_{Ca}
20
0
-20
-40
-60
-80
*
***
Baseline
Pyr3 (10μM)
Pyr3+TAK (10μM)
Pyr3+TAK (100μM)
